# (1*E*,4*E*)-1-(3-Nitro­phen­yl)-5-phenyl­penta-1,4-dien-3-one

**DOI:** 10.1107/S1600536811052548

**Published:** 2011-12-10

**Authors:** S. Samshuddin, Ray J. Butcher, Mehmet Akkurt, B. Narayana, B. K. Sarojini, H. S. Yathirajan

**Affiliations:** a Department of Studies in Chemistry, Mangalore University, Mangalagangotri, Mangalore 574 199, India; b Department of Chemistry, Howard University, 525 College Street NW, Washington, DC 20059, USA; c Department of Physics, Faculty of Sciences, Erciyes University, 38039 Kayseri, Turkey; d Department of Chemistry, P. A. College of Engineering, Nadupadav, Mangalore-574 153, India; e Department of Studies in Chemistry, University of Mysore, Manasagangotri, Mysore 570 006, India

## Abstract

In the title compound, C_17_H_13_NO_3_, the dihedral angle between the benzene rings is 31.21 (5)°. In the crystal, inversion dimers linked by pairs of C—H⋯O hydrogen bonds occur. A C—H⋯π inter­action is also indicated.

## Related literature

For the pharmacological importance of chalcones and bis­ chalcones, see: Sarojini *et al.* (2006 ▶); Dhar (1981 ▶); Dimmock *et al.* (1999 ▶); Satyanarayana *et al.* (2004 ▶). For our work on synthesis of different derivatives of chalcones, see: Baktır *et al.* (2011 ▶); Fun *et al.* (2010 ▶); Jasinski *et al.* (2010 ▶); Samshuddin *et al.* (2011*a* ▶,*b* ▶,*c* ▶). For related structures, see: Butcher *et al.* (2006*a* ▶,*b* ▶; 2007*a* ▶,*b* ▶,*c* ▶); Harrison *et al.* (2006 ▶); Hu *et al.* (2004 ▶); Fischer *et al.* (2007 ▶); Patil *et al.* (2007 ▶); Zhao *et al.* (2007 ▶).
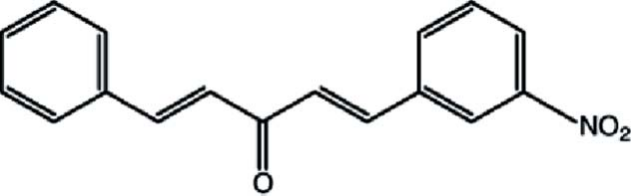


## Experimental

### 

#### Crystal data

C_17_H_13_NO_3_
*M* *_r_* = 279.28
Monoclinic, 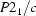

*a* = 11.9806 (6) Å
*b* = 9.8955 (4) Å
*c* = 12.5562 (7) Å
β = 114.992 (7)°
*V* = 1349.21 (14) Å^3^
*Z* = 4
Mo *K*α radiation
μ = 0.10 mm^−1^
*T* = 293 K
0.44 × 0.34 × 0.08 mm

#### Data collection

Oxford Diffraction Xcalibur Ruby Gemini diffractometer
Absorption correction: multi-scan (*CrysAlis RED*; Oxford Diffraction, 2007 ▶) *T*_min_ = 0.969, *T*_max_ = 1.000
7835 measured reflections
3746 independent reflections
2453 reflections with *I* > 2σ(*I*)
*R*_int_ = 0.033

#### Refinement

*R*[*F* ^2^ > 2σ(*F* ^2^)] = 0.055
*wR*(*F* ^2^) = 0.128
*S* = 1.02
3746 reflections
190 parameters
H-atom parameters constrained
Δρ_max_ = 0.25 e Å^−3^
Δρ_min_ = −0.26 e Å^−3^

### 

Data collection: *CrysAlis PRO* (Oxford Diffraction, 2007 ▶); cell refinement: *CrysAlis PRO*; data reduction: *CrysAlis RED* (Oxford Diffraction, 2007 ▶); program(s) used to solve structure: *SHELXS97* (Sheldrick, 2008 ▶); program(s) used to refine structure: *SHELXL97* (Sheldrick, 2008 ▶); molecular graphics: *ORTEP-3* (Farrugia, 1997 ▶); software used to prepare material for publication: *WinGX* (Farrugia, 1999 ▶), *PARST* (Nardelli, 1983 ▶) and *PLATON* (Spek, 2009 ▶).

## Supplementary Material

Crystal structure: contains datablock(s) global, I. DOI: 10.1107/S1600536811052548/tk5031sup1.cif

Structure factors: contains datablock(s) I. DOI: 10.1107/S1600536811052548/tk5031Isup2.hkl

Supplementary material file. DOI: 10.1107/S1600536811052548/tk5031Isup3.cml

Additional supplementary materials:  crystallographic information; 3D view; checkCIF report

## Figures and Tables

**Table 1:** Hydrogen-bond geometry (Å, °) *Cg*1 is the centroid of the C12–C17 phenyl ring.

*D*—H⋯*A*	*D*—H	H⋯*A*	*D*⋯*A*	*D*—H⋯*A*
C2—H2*A*⋯O3^i^	0.93	2.59	3.412 (2)	147
C5—H5*A*⋯*Cg*1^ii^	0.93	2.62	3.3915 (19)	141

Symmetry codes: (i) 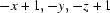; (ii) 
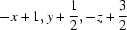
.

**Fig. 1.**
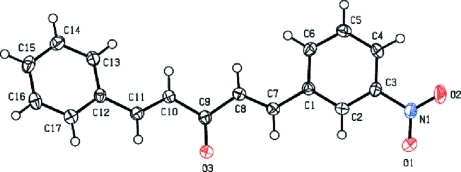
The molecular structure of the title compound, showing 50% probability displacement ellipsoids and the atom-numbering scheme.

**Fig. 2.**
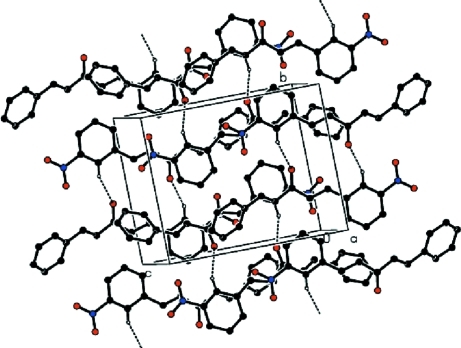
The crystal packing of the title compound, viewed approximately along the a axis. H atoms not involved in the intermolecular interactions (dashed lines) have been omitted for clarity.

## supplementary crystallographic information

### Comment

Chalcones are highly reactive substances of varied nature. They have been reported to possess many interesting pharmacological activities (Dhar, 1981) including anti-inflammatory, antimicrobial, antifungal, antioxidant, cytotoxic, antitumor and anticancer activities (Dimmock *et al.*, 1999; Satyanarayana *et al.*, 2004). Chalcones are also finding application as organic nonlinear optical materials (NLO) for their SHG conversion efficiency (Sarojini *et al.*, 2006). The basic skeleton of chalcones which possess α,β-unsaturated carbonyl group is useful for the synthesis of various biodynamic cyclic derivatives such as pyrazoline, benzodiazepine, 2,4,6-triaryl pyridine, isoxazoline and cyclohexenone derivatives (Samshuddin *et al.*, 2011*a*,*b*,*c*; Fun *et al.*, 2010; Jasinski *et al.*, 2010; Baktır *et al.*, 2011).

The crystal structures of some bis-chalcones *viz*., 2,6-bis(4-methoxybenzylidene)cyclohexanone (Butcher *et al.*, 2006*a*), 1,5-bis(4-chlorophenyl)penta-1,4-dien-3-one (Butcher *et al.*, 2006*b*), 1,5-bis(3,4-dimethoxyphenyl)penta-1,4-dien-3-one (Butcher *et al.*, 2007*a*), 1,5-bis(4-fluorophenyl)penta-1,4-dien-3-one (Butcher *et al.*, 2007*b*), 2,5-bis(3,4-dimethoxybenzylidene)cyclopentanone (Butcher *et al.*, 2007*c*), 1,5-bis(4-methoxyphenyl)penta-1,4-dien-3-one (Harrison *et al.*, 2006), 2,4-dimethyl-1,5-diphenylpenta-1,4-dien-3-one (Hu *et al.*, 2004) have been reported. In continuation of our work on synthesis of chalcone derivatives, the title compound (I) was prepared and its crystal structure is reported.

In the title molecule (I), (Fig. 1), bond lengths and angles are comparable to closely related structures (Patil *et al.*, 2007; Zhao *et al.*, 2007; Fischer *et al.*, 2007; Butcher *et al.* (2006*a*,*b*, 2007*a*,*b*,*c*); Harrison *et al.* (2006); Hu *et al.* (2004). The least-squares plane through the C7–C11/O3 group makes dihedral angles of 8.22 (6) and 32.14 (6)° with the C1–C6 benzene ring and the C12–C17 phenyl ring, respectively. The dihedral angle between these rings is 31.21 (5)° and the nitro group at C3 lies close to the C1–C6 ring plane, with O1–N1–C3–C2 and O2–N1–C3–C4 torsion angles of 6.3 (3) and 7.4 (3)°, respectively.

The molecular packing of (I) shown in Fig. 2 is stabilized by C—H···O interactions (Table 1), which lead to the formation of a centrosymmetric dimer, and is further consolidated by C—H···π interactions (Table 1) involving the C12–C17 phenyl ring.

### Experimental

Synthesis of title compound was carried out by stirring a mixture of benzylidene acetone (1.46 g, 0.01 mol) and 3-nitrobenzaldehyde (1.51 g, 0.01 mol) in 40 ml of ethanolic sodium hydroxide at 278–283 K for 3 h. The precipitate was collected by filtration and purified by recrystallization from ethanol. The single crystal was grown from 1,4-dioxane by the slow evaporation method and yield of the compound was 80%. (*M*.pt. 414 K).

### Refinement

All H atoms were positioned geometrically (C—H = 0.93 Å) and refined as riding, with *U*_iso_(H) = 1.2*U*_eq_(C).

### Crystal data

**Table:**

C_17_H_13_NO_3_	*F*(000) = 584
*M**_r_* = 279.28	*D*_x_ = 1.375 Mg m^−^^3^
Monoclinic, *P*2_1_/*c*	Mo *K*α radiation, λ = 0.71073 Å
Hall symbol: -P 2ybc	Cell parameters from 2209 reflections
*a* = 11.9806 (6) Å	θ = 3.1–30.9°
*b* = 9.8955 (4) Å	µ = 0.10 mm^−^^1^
*c* = 12.5562 (7) Å	*T* = 293 K
β = 114.992 (7)°	Plate, colourless
*V* = 1349.21 (14) Å^3^	0.44 × 0.34 × 0.08 mm
*Z* = 4	

### Data collection

**Table:**

Oxford Diffraction Xcalibur Ruby Gemini diffractometer	3746 independent reflections
Radiation source: Enhance (Mo) X-ray Source	2453 reflections with *I* > 2σ(*I*)
graphite	*R*_int_ = 0.033
Detector resolution: 10.5081 pixels mm^-1^	θ_max_ = 30.9°, θ_min_ = 3.3°
ω scans	*h* = −17→15
Absorption correction: multi-scan (*CrysAlis RED*; Oxford Diffraction, 2007)	*k* = −13→14
*T*_min_ = 0.969, *T*_max_ = 1.000	*l* = −11→18
7835 measured reflections	

### Refinement

**Table:**

Refinement on *F*^2^	Primary atom site location: structure-invariant direct methods
Least-squares matrix: full	Secondary atom site location: difference Fourier map
*R*[*F*^2^ > 2σ(*F*^2^)] = 0.055	Hydrogen site location: inferred from neighbouring sites
*wR*(*F*^2^) = 0.128	H-atom parameters constrained
*S* = 1.02	*w* = 1/[σ^2^(*F*_o_^2^) + (0.047*P*)^2^ + 0.2703*P*] where *P* = (*F*_o_^2^ + 2*F*_c_^2^)/3
3746 reflections	(Δ/σ)_max_ < 0.001
190 parameters	Δρ_max_ = 0.25 e Å^−^^3^
0 restraints	Δρ_min_ = −0.26 e Å^−^^3^

### Special details

**Table:**

Geometry. Bond distances, angles *etc*. have been calculated using the rounded fractional coordinates. All su's are estimated from the variances of the (full) variance-covariance matrix. The cell e.s.d.'s are taken into account in the estimation of distances, angles and torsion angles
Refinement. Refinement on *F*^2^ for ALL reflections except those flagged by the user for potential systematic errors. Weighted *R*-factors *wR* and all goodnesses of fit *S* are based on *F*^2^, conventional *R*-factors *R* are based on *F*, with *F* set to zero for negative *F*^2^. The observed criterion of *F*^2^ > σ(*F*^2^) is used only for calculating -*R*-factor-obs *etc*. and is not relevant to the choice of reflections for refinement. *R*-factors based on *F*^2^ are statistically about twice as large as those based on *F*, and *R*-factors based on ALL data will be even larger.

### Fractional atomic coordinates and isotropic or equivalent isotropic displacement parameters (Å^2^)

**Table:**

	*x*	*y*	*z*	*U*_iso_*/*U*_eq_	
O1	0.17385 (11)	0.14120 (12)	0.04941 (10)	0.0346 (4)	
O2	0.11627 (15)	0.33639 (14)	−0.03094 (11)	0.0526 (5)	
O3	0.54296 (11)	0.04578 (11)	0.67363 (10)	0.0288 (4)	
N1	0.16833 (13)	0.26465 (15)	0.05501 (12)	0.0291 (4)	
C1	0.34756 (14)	0.30928 (16)	0.37830 (13)	0.0208 (4)	
C2	0.29603 (14)	0.25023 (16)	0.26714 (13)	0.0211 (4)	
C3	0.22682 (14)	0.32911 (16)	0.17068 (13)	0.0226 (5)	
C4	0.20852 (15)	0.46592 (17)	0.17935 (14)	0.0248 (5)	
C5	0.26174 (15)	0.52505 (17)	0.28955 (14)	0.0247 (5)	
C6	0.32965 (15)	0.44777 (16)	0.38746 (14)	0.0242 (5)	
C7	0.41680 (14)	0.22370 (16)	0.48051 (14)	0.0225 (5)	
C8	0.46170 (14)	0.26055 (16)	0.59296 (14)	0.0227 (5)	
C9	0.53494 (14)	0.16722 (16)	0.68958 (14)	0.0225 (5)	
C10	0.60212 (14)	0.23207 (16)	0.80546 (13)	0.0221 (5)	
C11	0.70265 (14)	0.17661 (16)	0.88844 (13)	0.0226 (5)	
C12	0.78098 (14)	0.23472 (16)	1.00356 (13)	0.0211 (4)	
C13	0.74719 (15)	0.34930 (17)	1.04775 (14)	0.0256 (5)	
C14	0.82268 (16)	0.39866 (17)	1.15834 (15)	0.0296 (5)	
C15	0.93264 (16)	0.33433 (18)	1.22672 (15)	0.0307 (5)	
C16	0.96719 (15)	0.22128 (18)	1.18395 (15)	0.0288 (5)	
C17	0.89256 (15)	0.17173 (17)	1.07295 (14)	0.0258 (5)	
H2A	0.30800	0.15890	0.25790	0.0250*	
H4A	0.16180	0.51670	0.11310	0.0300*	
H5A	0.25180	0.61710	0.29780	0.0300*	
H6A	0.36420	0.48870	0.46100	0.0290*	
H7A	0.43070	0.13480	0.46530	0.0270*	
H8A	0.44660	0.34770	0.61140	0.0270*	
H10A	0.57350	0.31380	0.82090	0.0260*	
H11A	0.72550	0.09230	0.87160	0.0270*	
H13A	0.67340	0.39290	1.00270	0.0310*	
H14A	0.79940	0.47530	1.18680	0.0360*	
H15A	0.98280	0.36730	1.30110	0.0370*	
H16A	1.04090	0.17790	1.22970	0.0350*	
H17A	0.91710	0.09590	1.04460	0.0310*	

### Atomic displacement parameters (Å^2^)

**Table:**

	*U*^11^	*U*^22^	*U*^33^	*U*^12^	*U*^13^	*U*^23^
O1	0.0408 (8)	0.0265 (6)	0.0284 (7)	−0.0001 (6)	0.0068 (6)	−0.0062 (6)
O2	0.0800 (11)	0.0377 (8)	0.0184 (7)	−0.0015 (8)	−0.0003 (7)	0.0065 (6)
O3	0.0347 (7)	0.0216 (6)	0.0241 (6)	0.0027 (5)	0.0066 (5)	−0.0001 (5)
N1	0.0322 (8)	0.0298 (8)	0.0206 (7)	−0.0031 (7)	0.0066 (6)	−0.0004 (7)
C1	0.0195 (7)	0.0224 (8)	0.0202 (8)	0.0009 (6)	0.0082 (7)	0.0004 (7)
C2	0.0216 (7)	0.0183 (7)	0.0243 (8)	0.0010 (6)	0.0107 (7)	0.0001 (7)
C3	0.0240 (8)	0.0249 (8)	0.0171 (8)	−0.0039 (7)	0.0070 (7)	−0.0009 (7)
C4	0.0270 (8)	0.0251 (8)	0.0211 (8)	0.0029 (7)	0.0091 (7)	0.0067 (7)
C5	0.0307 (9)	0.0199 (8)	0.0270 (9)	0.0024 (7)	0.0156 (7)	0.0023 (7)
C6	0.0278 (8)	0.0259 (8)	0.0199 (8)	−0.0011 (7)	0.0112 (7)	−0.0032 (7)
C7	0.0216 (8)	0.0207 (8)	0.0226 (8)	0.0013 (7)	0.0068 (7)	−0.0001 (7)
C8	0.0228 (8)	0.0211 (8)	0.0230 (8)	0.0016 (7)	0.0085 (7)	−0.0001 (7)
C9	0.0204 (8)	0.0240 (8)	0.0222 (8)	−0.0003 (7)	0.0081 (7)	0.0012 (7)
C10	0.0253 (8)	0.0207 (8)	0.0192 (8)	0.0012 (7)	0.0084 (7)	0.0014 (7)
C11	0.0268 (8)	0.0206 (8)	0.0215 (8)	−0.0006 (7)	0.0112 (7)	0.0010 (7)
C12	0.0223 (8)	0.0227 (8)	0.0177 (7)	−0.0028 (7)	0.0079 (7)	0.0029 (7)
C13	0.0239 (8)	0.0259 (8)	0.0238 (8)	−0.0001 (7)	0.0071 (7)	0.0027 (7)
C14	0.0334 (9)	0.0262 (9)	0.0274 (9)	−0.0039 (8)	0.0111 (8)	−0.0043 (8)
C15	0.0310 (9)	0.0338 (10)	0.0223 (9)	−0.0092 (8)	0.0064 (8)	−0.0038 (8)
C16	0.0209 (8)	0.0352 (10)	0.0253 (9)	−0.0005 (7)	0.0050 (7)	0.0049 (8)
C17	0.0251 (8)	0.0264 (8)	0.0255 (8)	0.0006 (7)	0.0104 (7)	0.0010 (7)

### Geometric parameters (Å, °)

**Table:**

O1—N1	1.2270 (19)	C13—C14	1.387 (2)
O2—N1	1.2202 (19)	C14—C15	1.386 (3)
O3—C9	1.2287 (19)	C15—C16	1.378 (3)
N1—C3	1.465 (2)	C16—C17	1.389 (2)
C1—C2	1.394 (2)	C2—H2A	0.9300
C1—C6	1.399 (2)	C4—H4A	0.9300
C1—C7	1.467 (2)	C5—H5A	0.9300
C2—C3	1.384 (2)	C6—H6A	0.9300
C3—C4	1.383 (2)	C7—H7A	0.9300
C4—C5	1.385 (2)	C8—H8A	0.9300
C5—C6	1.383 (2)	C10—H10A	0.9300
C7—C8	1.332 (2)	C11—H11A	0.9300
C8—C9	1.482 (2)	C13—H13A	0.9300
C9—C10	1.479 (2)	C14—H14A	0.9300
C10—C11	1.333 (2)	C15—H15A	0.9300
C11—C12	1.468 (2)	C16—H16A	0.9300
C12—C13	1.395 (2)	C17—H17A	0.9300
C12—C17	1.397 (2)		
			
O1—N1—O2	123.25 (14)	C12—C17—C16	120.61 (16)
O1—N1—C3	118.38 (13)	C1—C2—H2A	120.00
O2—N1—C3	118.36 (14)	C3—C2—H2A	120.00
C2—C1—C6	118.32 (14)	C3—C4—H4A	121.00
C2—C1—C7	118.78 (14)	C5—C4—H4A	121.00
C6—C1—C7	122.89 (14)	C4—C5—H5A	120.00
C1—C2—C3	119.22 (15)	C6—C5—H5A	120.00
N1—C3—C2	118.73 (14)	C1—C6—H6A	119.00
N1—C3—C4	118.61 (14)	C5—C6—H6A	119.00
C2—C3—C4	122.65 (14)	C1—C7—H7A	117.00
C3—C4—C5	118.09 (15)	C8—C7—H7A	117.00
C4—C5—C6	120.27 (15)	C7—C8—H8A	119.00
C1—C6—C5	121.43 (15)	C9—C8—H8A	119.00
C1—C7—C8	126.52 (15)	C9—C10—H10A	119.00
C7—C8—C9	122.11 (15)	C11—C10—H10A	119.00
O3—C9—C8	122.43 (14)	C10—C11—H11A	117.00
O3—C9—C10	122.50 (15)	C12—C11—H11A	117.00
C8—C9—C10	115.02 (14)	C12—C13—H13A	120.00
C9—C10—C11	121.79 (15)	C14—C13—H13A	120.00
C10—C11—C12	126.80 (15)	C13—C14—H14A	120.00
C11—C12—C13	122.53 (15)	C15—C14—H14A	120.00
C11—C12—C17	118.99 (15)	C14—C15—H15A	120.00
C13—C12—C17	118.46 (14)	C16—C15—H15A	120.00
C12—C13—C14	120.55 (16)	C15—C16—H16A	120.00
C13—C14—C15	120.36 (16)	C17—C16—H16A	120.00
C14—C15—C16	119.65 (16)	C12—C17—H17A	120.00
C15—C16—C17	120.37 (17)	C16—C17—H17A	120.00
			
O1—N1—C3—C2	6.3 (3)	C1—C7—C8—C9	−177.47 (17)
O1—N1—C3—C4	−172.40 (17)	C7—C8—C9—O3	−11.3 (3)
O2—N1—C3—C2	−173.88 (18)	C7—C8—C9—C10	166.11 (17)
O2—N1—C3—C4	7.4 (3)	O3—C9—C10—C11	21.9 (3)
C6—C1—C2—C3	−1.6 (3)	C8—C9—C10—C11	−155.48 (17)
C7—C1—C2—C3	177.69 (17)	C9—C10—C11—C12	176.13 (16)
C2—C1—C6—C5	0.6 (3)	C10—C11—C12—C13	10.4 (3)
C7—C1—C6—C5	−178.66 (18)	C10—C11—C12—C17	−170.91 (18)
C2—C1—C7—C8	−173.22 (18)	C11—C12—C13—C14	178.32 (17)
C6—C1—C7—C8	6.1 (3)	C17—C12—C13—C14	−0.3 (3)
C1—C2—C3—N1	−177.19 (16)	C11—C12—C17—C16	−177.90 (16)
C1—C2—C3—C4	1.5 (3)	C13—C12—C17—C16	0.8 (3)
N1—C3—C4—C5	178.43 (17)	C12—C13—C14—C15	−0.3 (3)
C2—C3—C4—C5	−0.2 (3)	C13—C14—C15—C16	0.4 (3)
C3—C4—C5—C6	−0.8 (3)	C14—C15—C16—C17	0.0 (3)
C4—C5—C6—C1	0.6 (3)	C15—C16—C17—C12	−0.7 (3)

### Hydrogen-bond geometry (Å, °)

**Table:**

Cg1 is the centroid of the C12–C17 phenyl ring.

**Table:**

*D*—H···*A*	*D*—H	H···*A*	*D*···*A*	*D*—H···*A*
C2—H2A···O3^i^	0.93	2.59	3.412 (2)	147
C7—H7A···O3	0.93	2.54	2.858 (2)	100
C11—H11A···O3	0.93	2.57	2.8724 (19)	100
C5—H5A···Cg1^ii^	0.93	2.62	3.3915 (19)	141

Symmetry codes: (i) −*x*+1, −*y*, −*z*+1; (ii) −*x*+1, *y*+1/2, −*z*+3/2.
